# Crystal structures of multidrug efflux pump MexB bound with high-molecular-mass compounds

**DOI:** 10.1038/s41598-019-40232-2

**Published:** 2019-03-13

**Authors:** Keisuke Sakurai, Seiji Yamasaki, Kaori Nakao, Kunihiko Nishino, Akihito Yamaguchi, Ryosuke Nakashima

**Affiliations:** 1 Laboratory of Cell Membrane Structural Biology, Institute of Scientific and Industrial Research, Osaka University, Ibaraki, Osaka 567-0047 Japan; 2 Department of Biomolecular Science and Regulation, Institute of Scientific and Industrial Research, Osaka University, Ibaraki, Osaka 567-0047 Japan; 3 School of Pharmaceutical Sciences, Osaka University, Suita, Osaka 565-0871 Japan

## Abstract

RND-type multidrug efflux pumps have two voluminous multisite drug-binding pockets named the proximal and distal binding pocket. High- and low-molecular-mass drugs bind to these proximal and distal pocket, respectively. Here, we report the crystal structures of MexB of *Pseudomonas aeruginosa* bound with high-molecular-mass compounds. Contrary to the expectations, lauryl maltose neopentyl glycol (LMNG, MW 1,005), which is a surfactant larger than the proximal pocket-binding drugs, was found to bind to the distal pocket: one of the two hydrophobic alkyl chains was inserted into the hydrophobic pit, which is the binding site of the efflux pump inhibitor ABI-PP. LMNG is a substrate of the MexAB-OprM system and competitively inhibits the export of other substrates by this system. However, LMNG does not inhibit the export of other substrates by the inhibitor-binding-pit mutant F178W, which retains the export activity of LMNG. The crystal structure of this mutant suggested that the alkyl chain of LMNG could no longer be inserted into the pit because of steric hindrance. We also determined the crystal structure of MexB containing the high-molecular-mass compound neopentyl glycol derivative C7NG (MW 1,028), the binding site of which overlapped with LMNG in the distal pocket, indicating that whether a substrate binds to the distal or proximal pockets is controlled not only by its molecular weight but also by its individual molecular characteristic.

## Introduction

Antimicrobial resistance (AMR) has been a serious problem in modern chemotherapy since antibiotics were first administered. Currently, the battle between pathogenic bacteria and humanity enters a new stage consisting of the rise and spread of multidrug-resistant (MDR) bacteria amid a decline in the willingness of pharmaceutical companies to develop antibacterial drugs^1^. Newly emerging MDR gram-negative bacteria over-express RND-type multidrug efflux pumps^2,3^, which cause resistance against an extraordinarily wide range of antibiotics by a single factor^4^. RND-type exporters are part of  a tripartite complex, composed of an outer membrane channel, an inner membrane MDR pump and an adaptor protein^5^. The crystal structures of each component were solved^6–8^, but the determination of tripartite structures by X-ray crystallography has not succeeded. Instead of the crystal structure, the cryo-EM structures of the complex have been reported^9–11^. Among the three components of the complex, active energy coupling and substrate recognition are performed by the inner membrane pump^12^. We first determined the crystal structure of one of the inner membrane MDR pumps, AcrB^7^. Since then, we have revealed the molecular mechanism of multidrug efflux and multisite recognition by determining the substrate-binding structures of AcrB^13–15^. MDR pumps are characterized by the difficult in the identification of most bound drugs in their co-crystal structures^16^. Until now, there are only a few examples of substrate- and inhibitor-bound structures of the physiologically meaningful asymmetrical structures of MDR pumps^11,13–15,17–19^. For some reason, there are reports of substrate-bound structures of three-fold symmetrical trimers of MDR pumps^20–23^, while the functionally rotating mechanism of the drug efflux mediated by AcrB^14,24–30^ is hard to study because of these three-drug-molecule-bound symmetrical trimer structures. If such a symmetrical structure exists *in vivo*, it may be a resting form^16^. The difficulty in identifying drug-bound structures may be due to multisite drug binding^16,18^ and drug oscillation between binding sites during transport^16^. Although the range of drugs exported by MDR pumps is very wide and vague, the recognition of some drugs and inhibitors is strictly specific^13^. To reveal the principle of this broad recognition of substrates with strict specificity, we must determine more substrate-binding structures of MDR pumps.

There are two distinct multisite drug-binding pockets; the proximal and distal binding pocket, in MDR pumps^15^. In the distal binding pocket (DBP), there is a specific efflux pump inhibitor-binding hydrophobic pit for pyridopyrimidine and pyranopyridine derivatives^13,19^ branched from the substrate translocation pathway. The strong affinity of these inhibitors to the pit is thought to be the foundation of their inhibitory power^13^. Recently, a third binding site located at the transmembrane region near the periplasmic surface was also reported^31^. The presence of multiple binding sites and multiple pathways^16,21,32,33^ is likely related to the recognition of diverse substrates. We reported in our previous paper that high-molecular-mass drugs (HMMDs) and low-molecular-mass drugs (LMMDs) prefer the proximal binding pocket (PBP) and the DBP, respectively^15^. Here we show drug-bound structures of MexB from *P*. *aeruginosa* with high-molecular-mass compounds. Unexpectedly, these compounds bound to the DBP despite their high molecular mass. Part of their alkyl chains was inserted into the inhibitor-binding pit, and their competitive inhibitory activities to other drug efflux involves the insertion of their alkyl chain into the inhibitor-binding pit.

## Results

### Crystal structure of MexB bound with Lauryl maltose neopentyl glycol (LMNG)

**Figure 1:**
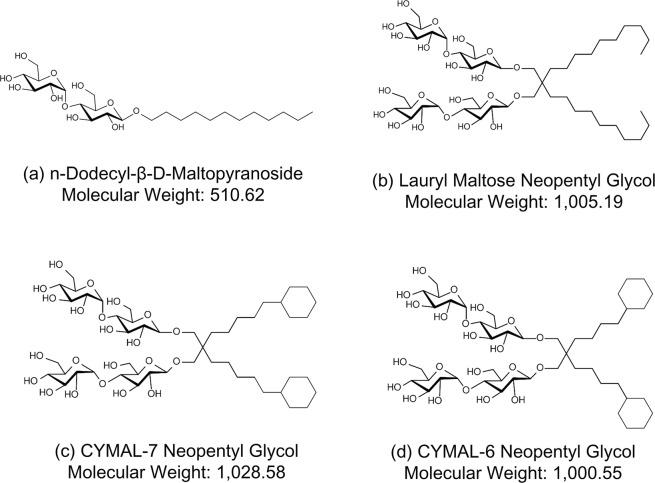
Chemical structures of n-dodecyl-β-D-maltopyranoside (DDM), lauryl maltose neopentyl glycol (LMNG), CYMAL-7 neopentyl glycol (C7NG), and CYMAL-6 neopentyl glycol (C6NG) used in this experiment.

Unlike the main RND transporter from *Escherichia coli* AcrB, bound drugs have not been identified in MexB crystal structures. Regardless of MexB crystalized with or without drugs, the DBP of MexB was occupied by a detergent molecule DDM^13,17^ (Fig. 1a). In order to identify bound drugs, we exchanged DDM by the large molecular weight detergent LMNG (Fig. 1b), which has a molecular weight almost twice as much as DDM. The molecular weight is not only higher than that of LMMDs (e.g. minocycline and doxorubicin), but is also higher than that of HMMDs (e.g. rifampicin and erythromycin). We expected that LMNG may not bind to DBP and may not disturb drug binding to the DBP.

**Figure 2:**
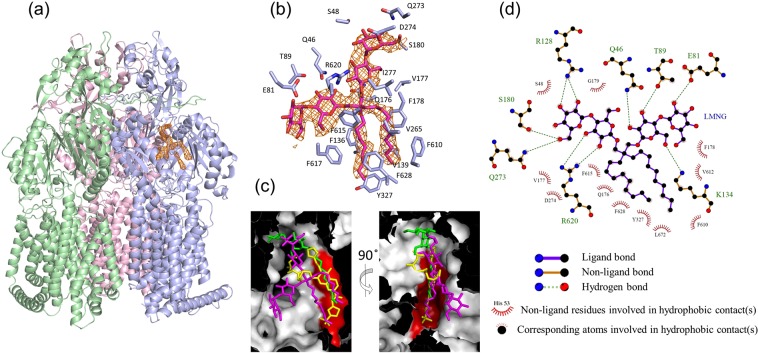
Crystal structure of LMNG-bound MexB. (**a**) Whole trimer structure of MexB (ribbon model) bound with LMNG (electron density map). Binding, extrusion and access protomers are shown in blue, pink and green, respectively. The LMNG-binding site is shown by the electron density calculated as an *F*o*-F*c omit map contoured at 2.8 σ (orange mesh). (**b**) Close-up view of the LMNG-binding site. Electron density of LMNG (orange mesh) overlapped with stick models of LMNG (pink). (**c**) Overlapping view of the bound LMNG (pink), DDM (green, PDB ID: 3W9I) and ABI-PP (yellow, PDB ID: 3W9J) shown in the cut view of the surface model of the distal pocket in the binding monomer. The inhibitor binding hydrophobic pit is shown by red surface. (**d**) The 2D representation of the interaction between LMNG and MexB (PDB ID: 6IIA) was drawn using *LigPlot*^+^^40^.

Surprisingly, we clearly detected the electron density of bound LMNG in the DBP of the MexB crystal structure, regardless whether or not drug was included in the crystallization medium. Figure 2a,b show the LMNG-bound structure of MexB. The binding site is composed of the PN1, PN2 and PC1 subdomains^7^ of the binding monomer of MexB (Fig. 2a,b). The hydroxymethyl group and the eight hydroxy groups of the glucose moieties of LMNG form hydrogen bonds with the side chains of Gln46, Glu81, Thr89, Arg128, Lys134, Ser180, Gln273 and Arg620 (Fig. 2d). The binding site of LMNG partially overlaps with the ABI-PP and DDM binding sites (Fig. 2c). One of the alkyl chains of LMNG is inserted into the inhibitor-binding hydrophobic pit^13^ (Fig. 2c). The strong interaction between bound ABI-PP and the wall of the hydrophobic pit is considered to be the cause of its inhibitory action^13^. The other alkyl chain of LMNG is elongated parallel in the space above the inhibitor-binding pit. Two maltose moieties are oppositely elongated towards the exit and entrance of the DBP (Supplementary Fig. S1). As a result, the LMNG molecule spreads throughout the DBP space, indicating that the DBP has sufficient space to accommodate a molecule with a molecular mass of more than 1,000, if it can specifically fit to the binding site. Although LMNG has a high molecular mass, the chemical structure is flexible, because of its many rotatable bonds. This highly flexible feature may also contribute to the ability of LMNG to traverse the narrow channel in order to reach the DBP. In addition, principal moments of inertia (PMI) analysis^34^ of RND pump substrates were plotted (Supplementary Fig. S2). This analysis can visualize structural diversity by categorizing a molecular shape into distinct topologies: rod-, sphere- and disc-like character. It shows that DBP-binding drugs share rod-like features. LMNG is a compound which is able to form a rod-like structure. On the other hand, the PBP-binding substrates do not have this feature. The peristaltic motion, including the swinging of the switch-loop may be necessary to translocate the PBP-binding drugs into DBP and, once they enter into the DBP, do not flow back and are consequently occluded in the DBP^13^.

### LMNG as a substrate of MexB and a competitive inhibitor of MexAB-OprM-mediated drug efflux

**Figure 3:**
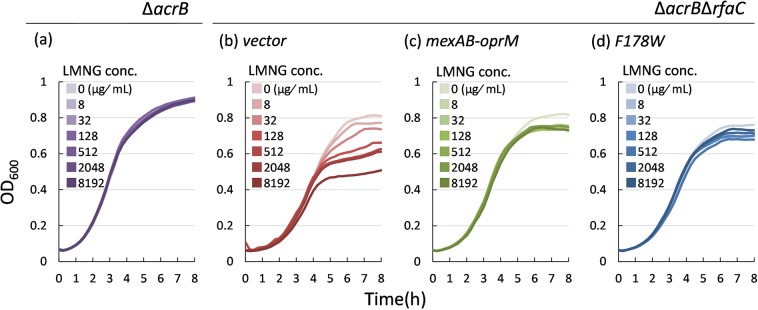
Antimicrobial activity of LMNG measured by the effect on growth curves of *Salmonella enterica* serovar typhimurium. (**a**) Δ*acrB* (NKS148). (**b**) Δ*acrB*Δ*rfaC* transduced with the vector (pMMB67HE) (NKS1421). (**c**) Δ*acrB*Δ*rfaC* expressing MexAB-OprM (NKS1422). (**d**) Δ*acrB*Δ*rfaC* expressing MexAB(F178W)-OprM (NKS1423).

LMNG does not affect the growth of *P*. *aeruginosa* (data not shown) probably due to the low permeability of the outer membrane of *P*. *aeruginosa*. To reveal the properties of LMNG as a substrate of MexB, *acrB*-deficient *Salmonella enterica* serovar typhimurium was transduced with *mexAB-oprM*-encoding plasmids. Figure 3 shows the effect of LMNG on the growth of *S*. *enterica*. The growth of the *acrB*-deficient *S*. *enterica* cells was not affected by LMNG (Fig. 3a). However, the growth of the *acrB*-deficient rough mutant (Δ*acrB*Δ*rfaC*) cells^35^ was significantly inhibited by LMNG in a concentration-dependent manner (Fig. 3b), indicating that the polysaccharide chains of LPS in the outer membrane hinder the penetration and antibiotic action of the LMNG molecules. When MexAB-OprM was expressed, the growth of the Δ*acrB*Δ*rfaC* mutant was no longer affected by LMNG (Fig. 3c), clearly indicating that LMNG is exported by MexB. The small initial decrease in growth by the addition of 8 μg/mL LMNG (Fig. 3c) is probably due to the effect of disruption of the outer membrane surface by the surfactant action of LMNG. Because the CMC value of LMNG is 10 μg/mL (in water) according to the product description, the degree of the surfactant action was almost saturated at 8 μg/mL. Phe178 is the residue located at the middle of the inhibitor-binding pit and is important for inhibitor-binding, while the F178W mutant (which partly closes the pit and prevents the binding of inhibitor ABI-PP in AcrB) still retains drug export activity^13^. In this experiment, MexA-MexB(F178W)-OprM was also expressed in *S*. *enterica*. The expression level of this mutant was the same as that of the wild type (Supplementary Fig. S3). The growth of MexB(F178W)-expressing cells was not significantly affected by LMNG (Fig. 3d), indicating that the mutant retains its LMNG-export activity. The very slight LMNG-dose-dependent decrease in growth of MexB(F178W) may be caused by a decrease in the export activity by the mutation itself.

**Figure 4:**
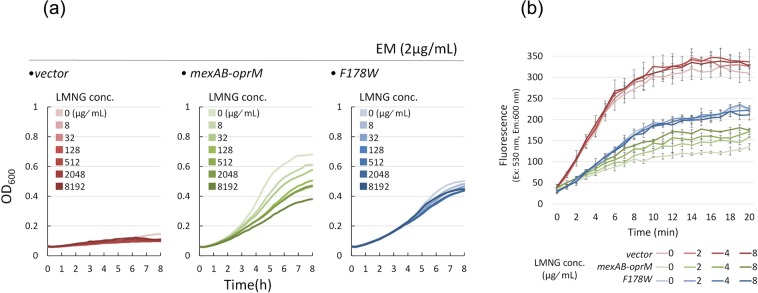
Inhibitory effect of LMNG on MexAB-OprM-mediated drug efflux in the rough mutant of *acrB*-deficient *S*. *enterica*. (**a**) The effect of LMNG on the growth of cells in the presence of a low concentration (2 μg/ml) of erythromycin. Left panel: the strain transduced with the vector, middle panel: the strain expressing MexA-MexB-OprM, right panel: the strain expressing MexA-MexB(F178W)-OprM. (**b**) The effect of LMNG on the MexAB-OprM-mediated prevention of ethidium bromide accumulation in the Δ*acrB*Δ*rfaC* mutant of *S*. *enterica*. The strain transduced with the vector (red), the strain expressing MexA-MexB-OprM (green) and the strain expressing MexA-MexB(F178W)-OprM (blue) were used. Then, 10 µM ethidium bromide and the indicated amount of LMNG were added.

Figure 4 shows the competitive inhibition by LMNG against erythromycin (EM) and ethidium bromide (EtBr) MexB-mediated export. In the presence of 2 μg/mL EM, the Δ*acrB*Δ*rfaC* cells did not show any growth regardless of the presence or absence of LMNG (Fig. 4a, left panel), while the MexAB-OprM-expressing cells were able to grow in the presence of 2 μg/mL EM. The growth was significantly decreased by the addition of LMNG in a dose-dependent manner (Fig. 4a, middle panel), indicating that LMNG competitively inhibits EM extrusion by MexB as these same concentrations of LMNG alone did not affect the growth at all (Fig. 3c). As for the MexB(F178W)-expressing cells, the presence of 2 μg/mL EM did not inhibit the growth of the cells, however, the overall growth was lower than that of the wild-type expressing cells due to the low EM extrusion activity of the mutant MexB. Nevertheless, contrary to the wild-type expressing cells, the growth of the mutant-expressing cells was essentially not inhibited by LMNG (Fig. 4a, right panel).

Next, we tested the competitive inhibition of LMNG against EtBr export. EtBr accumulates in the Δ*acrB*Δ*rfaC* cells of the *S*. *enterica* strain and yields fluorescence by intercalation into the chromosomal DNA (Fig. 4b, red lines). The EtBr accumulation in the absence of the MDR pump was almost not affected by the addition of LMNG. In the case of the wild-type MexAB-OprM-expressing cells, EtBr accumulation was very low as the drug export activity of MexB prevented EtBr from entering the cells (Fig. 4b, pale green line). When LMNG was added, the prevention became weaker, and the degree of EtBr accumulation increased depending on the LMNG concentration (Fig. 4b, green lines), indicating that LMNG competitively inhibits MexB-mediated EtBr export. The MexB(F178W)-expressing cells showed an intermediate level of EtBr accumulation, between that of the Δ*acrB*Δ*rfaC* cells and the wild-type MexB-expressing cells, due to the lower but significant EtBr-export activity of MexB(F178W) (Fig. 4b, pale blue line). Contrary to the wild type, the EtBr export activity of the mutant was not affected by LMNG addition at all (Fig. 4b, blue lines). In summary, both EM and EtBr efflux are competitively inhibited by LMNG in wild-type MexB but not inhibited in MexB(F178W).

We obtained similar results with clarithromycin (CAM), ciprofloxacin (CPFX), and azithromycin (AZM) in a growth curve assay (Supplementary Fig. S4a) and also berberine (BER) in a fluorescence accumulation assay (Supplementary Fig. S4b). The export of these drugs by MexB was inhibited by LMNG in wild-type MexB, but not in the F178W mutant. On the other hand, the export activity of doxorubicin (DXR), minocycline (MINO) and rhodamine 6 G (R6G) was not significantly affected by LMNG in both wild-type MexB and mutant MexB (Supplementary Fig. S5). These drugs not affected by LMNG are the drugs of which the DBP-binding structures of AcrB were previously reported^14,19^. While the affinity of LMNG to MexB could not be measured directly, it should be estimated to be low when considering the concentrations required for the inhibition of the EM export (Fig. 4a, middle panel). Thus, it might not inhibit the drugs clearly binding to the DBP. The drugs inhibited by LMNG in wild type MexB, are drugs that bind to the PBP in AcrB or drugs of which binding structures were not determined in AcrB nor MexB. The binding affinity of these drugs to the DBP should be very low. LMNG might only inhibit the export of DBP-not-bound or DBP-very-low-affinity-bound drugs.

### Crystal structure of MexB(F178W) co-crystalized with LMNG

We determined the crystal structure of MexB(F178W) co-crystalized with LMNG (Supplementary Fig. S6). Supplementary Fig. S6a shows a close-up view of the DBP in the MexB(F178W) co-crystal with LMNG. The angle and range of view are the same as those in Fig. 2b. Although slight positive electron densities were observed in the *F*o-*F*c omit map, it is not sufficient to identify bound LMNG. Supplementary Fig. S6b shows the LMNG molecule in the DBP, which was obtained by docking simulations using *Glide* (Schrödinger). The alkyl chain of LMNG, which was inserted into the inhibitor-binding pit in the wild-type MexB, was located outside the pit of MexB(F178W). The indole ring of the bulky tryptophan side chain was slightly protruded into the pit (Supplementary Fig. S6b) and likely interferes the insertion of the alkyl moiety of LMNG. In the ABI-PP-bound structure of MexB(F178W)^15^ the π-π interaction between the indole and pyridopyrimidine ring pushed the indole ring into the flat wall. However, the alkyl chain of LMNG was no longer able to overcome the steric hindrance by the indole ring and could not insert into the pit.

### Co-crystal structures of MexB with other high-molecular-mass neopentyl glycol (NG) derivatives CYMAL-7 neopentyl glycol (C7NG) and CYMAL-6 neopentyl glycol (C6NG)

**Figure 5:**
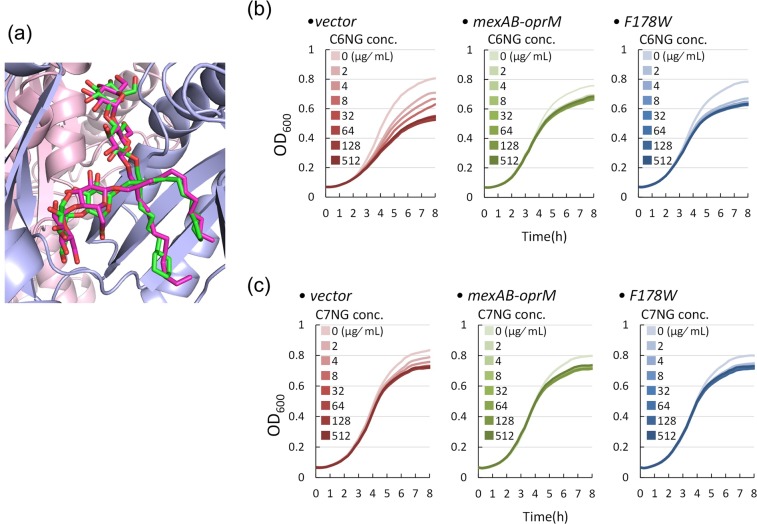
Crystal structure of the C7NG-binding site of MexB (**a**) and the effect of C6NG (**b**) and C7NG (**c**) on the growth of the Δ*acrB*Δ*rfaC* mutant of *S*. *enterica*. (**a**) Close-up view of the C7NG-binding site in MexB overlapped with bound LMNG. (**b**,**c**) The left panels indicate the strain transduced with the vector, the middle panels indicate the strain expressing MexA-MexB-OprM, and the right panels indicate the strain expressing MexA-MexB(F178W)-OprM.

To reveal whether it is a general rule or not that high-molecular-mass NG derivatives can bind in the DBP, we tried to determine the crystal structures of MexB with C7NG and C6NG. These molecules are analogues of LMNG, but the alkyl chains are terminally substituted with cyclohexane (Fig. 1c,d). Figure 5a shows the structure of C7NG-bound MexB. The electron density derived from C7NG was observed in the DBP (Supplementary Fig. S7). While the electron density was poor, one of the alkyl chains was inserted into the pit. The positions of the C7NG and the LMNG molecule almost completely overlapped in the binding site. On the other hand, we could not identify a bound C6NG molecule.

We measured the effect of C6NG and C7NG on the growth of the Δ*acrB*Δ*rfaC S*. *enterica* cells (Fig. 5b,c). C6NG showed growth inhibition against the strain without MDR pumps (Fig. 5b, left panel), but did not affect the growth of wild type MexB-expressing cells (Fig. 5b, middle panel) nor the F178W mutant-expressing cells (Fig. 5b right panel). The initial decrease in the growth at the minimum concentration of C6NG (2 μg/mL) is probably due to the surfactant action of C6NG causing outer membrane disruption, which is independent of the export of C6NG. Thus, similar to LMNG, C6NG is shown to be a substrate of MexB and MexB(F178W). On the other hand, the inhibitory activity of C7NG on the growth of Δ*acrB*Δ*rfaC* cells was very small (Fig. 5c, left panel). This result may be due to the low permeability of C7NG through the outer membrane, even in the rough mutants. Although there is no biochemical evidence which shows that C7NG is a substrate of MexB, because it does not obstruct growth of host bacteria and does not compete with the efflux activity of MexB (Fig. 5c), we argue that C7NG is a substrate of MexB from the fact that the C7NG binding crystal structure was obtained, and the fact that its NG-colleagues LMNG and C6NG are both substrates of MexB.

Finally, we measured the inhibitory effect of C6NG and C7NG on MexB-mediated EtBr export (Supplementary Fig. S8) by the method described in Fig. 4b. Although there are variations among experiments, the EtBr accumulation-prevention activity of the wild-type MexB is significantly inhibited by C6NG, while that of MexB(F178W) is not significantly inhibited, similar to LMNG. On the other hand, the inhibitory activity of C7NG was not observed, probably reflecting the low permeability of C7NG.

## Discussion

The results presented here show that substrates with a molecular mass greater than 1,000 can bind to the DBP if the binding site fits the substrate. Unlike DBP-binding LMMDs, such as MINO and DOX, LMNG and its related compounds are HMMDs and spread through the whole DBP space. One of the two alkyl chains is inserted into the hydrophobic inhibitor-binding pit. LMNG inhibits the export of EM, EtBr, CAM, CPFX, AZM and BER, but does not inhibit the export of DXR, MINO and R6G. When Phe178 was replaced by the bulky Trp residue, the acyl chain of LMNG could no longer be inserted into the inhibitor-binding pit in MexB(F178W) and the export of the drugs was no longer inhibited by LMNG.

Among the substrates which were inhibited by LMNG, EM is a PBP-binding drug^15^, and CAM, CPX, AZM and BER are the drugs of which bound structures were not detected in their co-crystals of MexB nor AcrB. In contrast, the drugs not inhibited by LMNG are the drugs of which DBP-binding structures were determined^14,19^. The former group of drugs are likely binding weakly or are merely occluded in the DBP. That is the reason why the export of these drugs are inhibited by LMNG. In our previous paper^33^ we reported that the channel 3-translocating drugs (CH3-drugs) such as EtBr are not competitively inhibited by DBP-binding drugs such as MINO and DOX, although we show here that EtBr and BER, which are CH3-drugs, are inhibited by LMNG. The difference is probably that the bound LMNG molecule occupies almost the entire DBP space in contrast to MINO and DOX, which occupy only a corner of the DBP^14^ and remain open the CH3 junction to the DBP^33^.

Nonetheless, the inhibitory activity of LMNG against the export of no- or weak-DBP-binding drugs was completely lost in the F178W mutant MexB. We reported in our previous paper that the F178W mutation causes the efflux pump inhibitor ABI-PP to lose its inhibitory activity in AcrB by the steric hindrance of the voluminous indole side chain in the inhibitor-binding pit, while the inhibitory activity was retained in MexB because there is still some space to facilitate the voluminous indole side chain into the pit wall of MexB^13^. The difference between ABI-PP and LMNG is the presence or absence of π-π interaction between the pyridopyrimidine ring of ABI-PP and the aromatic ring of the amino acid side chain at position 178. The π-π interaction might push the indole side chain of Trp178 into the pit wall in MexB. In contrast, since LMNG does not interact with the pit wall of the F178W mutant by strong interaction such as π-π interaction, the acyl chain did not overcome the steric hindrance. MexB(F178W), which was co-crystallized with LMNG, showed that the indole side chain of F178W is protruded into the pit and is likely to disturb the acyl chain of LMNG inserting into the pit. The docking model of LMNG in the DBP of MexB(F178W) calculated by *Glide* indicates that the acyl chain is pushed out of the pit.

The maximum calculated binding energy of LMNG to MexB(F178W) using *Glide* was −8.0 kcal/mol, which is only 60% compared to wild-type MexB (−13.4 kcal/mol). Although the binding energy calculated using *Glide* based on the DBP-bound structures without considering the path to reach the DBP does not show the actual affinity, the LMNG affinity to MexB should be reduced by the F178W mutation if the other conditions are the same. Thus, the close relationship between the insertion of a compound into the inhibitor-binding hydrophobic pit and its inhibitory activity for RND-type exporter-mediated export was also proved here in MexB.

On the other hand, LMNG did not inhibit the drugs which were clearly bound to the DBP. Although the binding energy of LMNG to the DBP in MexB calculated by *Glide* is higher (−13.4 kcal/mol) than those values for MINO and DOX (−5.6 and −5.2 kcal/mol in AcrB, respectively^13^). The values for MINO and DOX in MexB are not able to be calculated due to the absence of a MexB-bound structure for these drugs, however, the actual affinity of LMNG to MexB should be lower than MINO and DOX estimated from the very high inhibitory concentration of LMNG against EM export (Fig. 4a). For future research, it would be better if the actual affinity could be measured using purified MexB or using a reconstituted system, although no one has succeeded until now.

PMI analysis shows that the compound has a rod-like feature that can go through the narrow channel to reach the DBP without MexB’s peristaltic motion. The compound seems to be a DBP-binding substrate, which has a large distribution in the rod-like feature area in the PMI plot, despite its high molecular mass greater than those of the PBP-binding substrates. On the other hand, the PBP-binding substrates cannot go through the narrow channel because of their bulky structure with disc- and sphere-like features. The peristaltic motion of the RND pump may be required for these compounds to go through the narrow channel and to be translocated from the PBP to the DBP. Rhodamine 6G is the only exception which does not take a rod-like structure as a DBP-bound substrate. It can traverse channel 3 which enables the compound to bind to the DBP^33^.

In summary, multidrug recognition by RND transporters are mediated by complex multisite-binding mechanisms in the voluminous binding pockets of the transporters and the inhibitory activity of the substrates is deeply related to the insertion of a part of the compounds into the hydrophobic pit in the DBP. Our findings about the mechanism of substrate recognition, inhibition and the efflux pathways are supported by the studies of many investigators using computational simulations, including molecular dynamics calculations^36,37^ and the 3DRISM approach^38^.

## Methods

### Bacterial strains and growth conditions

The bacterial strains used in this study are listed in Supplementary Table S1. ∆*acrB* was derived from *S*. *enterica* ATCC 14028s^39^, and *rfaC*, one of the genes encoding LPS core biosynthetic proteins, was also disrupted^35^. The plasmids were introduced into different strains via electroporation, and the selection of desired strains was achieved by using appropriate antibiotics that were supplemented in the growth media. Cells were cultured at 37 °C in Luria-Bertani (LB) broth.

### Protein preparation and crystallization

Histidine-tagged MexB and mutant MexB(F178W) were expressed and purified as described previously^13^ with slight modifications. Protein was solubilized with 1.5% lauryl maltose neopentyl glycol (LMNG) or CYMAL-7 neopentyl glycol (C7NG) instead of n-dodecyl-β-D-maltopyranoside (DDM). Purified protein was concentrated in 10 mM Tris-HCl (pH 7.5), 50 mM NaCl and 0.05% detergent. MexB(F178W)/LMNG and MexB/C6NG samples were solubilized with DDM, which was replaced with LMNG or C6NG in the final step. Crystals were obtained from each sample using similar reservoir conditions^13^ with slight adjustments of pH and precipitant concentration. Cryoprotection was achieved by increasing the polyethylene glycol 400 concentration to 40% (v/v) in three steps. The crystals were flash frozen in liquid nitrogen.

#### Crystallographic analysis

Each data set was collected at the BL44XU beamline in SPring-8 with a MX300-HE detector (Rayonix) at 100 K. The diffraction images were indexed, integrated, scaled and merged using the *HKL2000* program package. Initial phasing of the crystals was performed by molecular replacement using the determined DDM-bound MexB structure (PDBID:3W9I) as the search model. The structures were refined and remodelled using the programs *REFMAC* (CCP4) and *COOT*, respectively. The *F*o-*F*c omit maps were calculated with coefficients of (*F*o-*F*c)exp(2π*i*α_non-substrate_), where α_non-substrate_ is the phase without LMNG or C7NG. The quality of the diffraction data and refinement statistics are provided in Supplementary Table S2. Each figure was drawn using *PyMOL* (Schrödinger).

### The effect of neopentyl glycol (NG) derivatives on bacterial growth

The cultures were diluted to OD_600nm_ = 0.1 in LB broth supplemented with the indicated concentrations of NG derivatives and/or 2 µg/mL erythromycin in a 96-well microtiter plate. Growth curves were plotted by measuring OD_600nm_ periodically with an Infinite M200 Pro (TECAN) microtiter plate reader. All microtiter wells contained 2.5% DMSO (final concentration), which was the solvent of the detergent stocks.

### Assay for inhibitory activity of NG derivatives on EtBr export

Cells were harvested when OD_600nm_ = 0.75 was reached, washed twice with efflux assay buffer (100 mM potassium phosphate (pH 7.5) and 5 mM MgSO_4_) and adjusted to a final OD_600nm_ of 36. After incubation on ice with NG derivatives for an hour in a black microtiter plate, EtBr was added to a final concentration of 10 µM. Preincubation is necessary for NG derivatives to sufficiently penetrate through the outer membrane. The fluorescence was measured by an SH-8100 reader (Corona Electric Co.) using λ_ex_ = 530 nm and λ_em_ = 600 nm. All the wells contained 2.5% DMSO (final concentration) as described above. Efflux assays were repeated at least three times.

### Shape diversity analysis (principal moments of inertia analysis)

1016, 1206, 1241, 1530, 1544, 550, 1416, 950 and 1204 conformations were generated for MBX3132, doxorubicin, erythromycin, LMNG, DDM, minocycline, ABI-PP, rifampicin and rhodamine 6G, respectively, using the program *MacroModel* (Schrödinger). Normalized ratios of principal moments of inertia (PMI) for each compound were calculated using Schrödinger Suite and plotted. Horizontal and vertical axis show normalized PMI ratios with I1/I3 (globularity) and I2/I3 (eccentricity; ECC), respectively.

### Binding affinity analysis

The binding affinity of each compound is calculated using the program *Glide* (Schrödinger) with no ligand sampling (score in place only).

## Supplementary information

Supplementary Figures

## Data Availability

The coordinate for LMNG-bound MexB has been deposited in the Protein Data Bank under accession number 6IIA.

## References

[1] Bush K (2011). Tackling antibiotic resistance. Nat. Rev. Microbiol..

[2] Piddock LJ (2006). Clinically relevant chromosomally encoded multidrug resistance efflux pumps in bacteria. Clin. Microbiol. Rev..

[3] Li XZ, Plésiat P, Nikaido H (2015). The challenge of efflux-mediated antibiotic resistance in Gram-negative bacteria. Clin. Microbiol. Rev..

[4] Schindler BD, Kaatz GW (2016). Multidrug efflux pumps of Gram-positive bacteria. Drug. Resist. Updat..

[5] Nikaido H (2009). Multidrug resistance in bacteria. Annu. Rev. Biochem..

[6] Koronakis V, Sharff A, Koronakis E, Luisi B, Hughes C (2000). Crystal structure of the bacterial membrane protein TolC central to multidrug efflux and protein export. Nature.

[7] Murakami S, Nakashima R, Yamashita E, Yamaguchi A (2002). Crystal structure of bacterial multidrug efflux transporter AcrB. Nature.

[8] Mikolosko J, Bobyk K, Zgurskaya HI, Ghosh P (2006). Conformational flexibility in multidrug efflux system protein AcrA. Structure.

[9] Du D (2014). Structure of the AcrAB-TolC multidrug efflux pump. Nature.

[10] Jeong H (2016). Pseudoatomic structure of the tripartite multidrug efflux pump AcrAB-TolC reveals the intermeshing cogwheel-like interaction between AcrA and TolC. Structure.

[11] Wang Z (2017). An allosteric transport mechanism for the AcrAB-TolC multidrug efflux pump. eLIFE.

[12] Elkins CA, Nikaido H (2002). Substrate specificity of the RND-type multidrug efflux pumps AcrB and AcrD of *Escherichia coli* is determined predominantly by two large periplasmic loops. J. Bacteriol..

[13] Nakashima R (2013). Structural basis for the inhibition of bacterial multidrug exporters. Nature.

[14] Murakami S, Nakashima R, Yamashita E, Matsumoto T, Yamaguchi A (2006). Crystal structures of a multidrug transporter reveal a functionally rotating mechanism. Nature.

[15] Nakashima R, Sakurai K, Yamasaki S, Nishino K, Yamaguchi A (2011). Structures of the multidrug exporter AcrB reveal a proximal multisite drug-binding pocket. Nature.

[16] Yamaguchi A, Nakashima R, Sakurai K (2015). Structural basis of RND-type multidrug exporters. Front. Microbial..

[17] Sennhauser G, Bukowska MA, Briand C, Grütter MG (2009). Crystal structure of the multidrug exporter MexB from *Pseudomonas aeruginosa*. J. Mol. Biol..

[18] Eicher T (2012). Transport of drugs by the multidrug transporter AcrB involves an access and a deep binding pocket that are separated by a switch-loop. Proc. Natl. Acad. Sci. USA.

[19] Sjuts H (2016). Molecular basis for inhibition of AcrB multidrug efflux pump by novel and powerful pyranopyridine derivatives. Proc. Natl. Acad. Sci. USA.

[20] Yu EW, McDermott G, Zgurskaya HI, Nikaido H, Koshland DE (2003). Structural basis of multiple drug-binding capacity of the AcrB multidrug efflux pump. Science.

[21] Yu EW, Aires JR, McDermott G, Nikaido H (2005). A periplasmic drug-binding site of the AcrB multidrug efflux pump: a crystallographic and site-directed mutagenesis study. J. Bacteriol..

[22] Drew D (2008). The structure of the efflux pump AcrB in complex with bile acid. Mol. Membr. Biol..

[23] Hung LW (2013). Crystal structure of AcrB complexed with linezolid at 3.5 Å resolution. J. Struct. Funct. Genomics.

[24] Seeger MA (2008). Engineered disulfide bonds support the functional rotation mechanism of multidrug efflux pump AcrB. Nat. Struct. Mol. Biol..

[25] Takatsuka Y, Nikaido H (2009). Covalently linked trimer of the AcrB multidrug efflux pump provides support for the functional rotating mechanism. J. Bacteriol..

[26] Feng Z, Hou T, Li Y (2012). Unidirectional peristaltic movement in multisite drug binding pockets of AcrB from molecular dynamic solutions. Mol. Biosyst..

[27] Schulz R, Vargiu AV, Collu F, Kleinekathöfer U, Ruggerone P (2010). Functional rotation of the transporter AcrB: insights into drug extrusion from simulations. PLoS Comput. Biol..

[28] Wang B, Weng J, Wang W (2015). Substrate binding accelerates the conformational transitions and substrate dissociation in multidrug efflux transporter AcrB. Front. Microbiol..

[29] Mishima H, Ohshima H, Yasuda S, Kinoshita M (2015). Statistical thermodynamics for functionally rotating mechanism of the multidrug efflux transporter AcrB. J. Phys. Chem..

[30] Sennhauser G, Amstutz P, Briand C, Storchenegger O, Grütter MG (2007). Drug export pathway of multidrug exporter AcrB revealed by DARPin inhibitors. PLoS Biol..

[31] Oswald C, Tam HK, Pos KM (2016). Transport of lipophilic carboxylates is mediated by transmembrane helix 2 in multidrug transporter AcrB. Nat. Commun..

[32] Husain F, Nikaido H (2010). Substrate path in the AcrB multidrug efflux pump of *Escherichia coli*. Mol. Microbiol..

[33] Zwama M (2018). Multiple entry pathways within the efflux transporter AcrB contribute to multidrug recognition. Nat. Commun..

[34] Sauer WHB, Schwarz MK (2003). Molecular Shape Diversity of Combinatorial Libraries: A Prerequisite for Broad Bioactivity. J. Chem. Inf. Comput. Sci..

[35] Yamasaki S, Nagasawa S, Fukushima A, Hayashi-Nishino M, Nishino K (2013). Cooperation of the multidrug efflux pump and lipopolysaccharides in the intrinsic antibiotic resistance of *Salmonella enterica* serovar Typhimurium. J. Antimicrob. Chemother..

[36] Zuo Z, Weng J, Wang W (2016). Insights into the Inhibitory Mechanism of D13-9001 to the Multidrug Transporter AcrB through Molecular Dynamics Simulations. J. Phys. Chem. B.

[37] Ramaswamy VK, Vargiu AV, Malloci G, Dreier J, Ruggerone P (2017). Molecular Rationale behind the Differential Substrate Specificity of Bacterial RND Multi-Drug Transporters. Scientific Reports.

[38] Imai T (2016). Functionality mapping on internal surfaces of multidrug transporter AcrB based on molecular theory of solvation: implications for drug efflux pathway. J. Phys. Chem. B.

[39] Fields PI, Swanson RV, Haidaris CG, Heffron F (1986). Mutants of *Salmonella typhimurium* that cannot survive within the macrophage are avirulent. Proc Natl Acad Sci USA.

[40] Laskowski RA, Swindells MB (2011). LigPlot+: multiple ligand-protein interaction diagrams for drug discovery. J. Chem. Inf. Model..

