# Supplementary material for: Crystal structures of multidrug efflux pump MexB bound with high-molecular-mass compounds
Source: Sci Rep. 2019 Mar 13;9:4359. doi: 10.1038/s41598-019-40232-2 (PMC6416280; doi:10.1038/s41598-019-40232-2)
Supplement: Supplementary file 1 — Supplementary Figures [file 41598_2019_40232_MOESM1_ESM.pdf]

Supplementary Information

Crystal structures of multidrug efflux pump MexB bound with high molecular mass compounds

Keisuke Sakurai[1], Seiji Yamasaki[2], Kaori Nakao[2,3], Kunihiko Nishino[2,3], Akihito Yamaguchi[1] and Ryosuke Nakashima[1‡]

[1]Laboratory of Cell Membrane Structural Biology and [2]Department of Biomolecular Science and Regulation, Institute of Scientific and Industrial Research, Osaka University, Ibaraki, Osaka567-0047, Japan, [3]School of Pharmaceutical Sciences, Osaka University, Suita, Osaka 565-0871, Japan,

[‡]Corresponding author. (email:nakashi@sanken.osaka-u.ac.jp)

Supplementary Table S1. *Salmonella enterica* serovar Typhimurium strains used in this experiment.

Strains	Characteristics	Source or reference
NKS148	Δ*acrB*	supplementary reference
NKS1279	Δ*acrB* Δ*rfaC*	Ref. 35
NKS1421	Δ*acrB* Δ*rfaC* /pMMB67HE	This study
NKS1422	Δ*acrB* Δ*rfaC* /pMMB67HE-*mexAB* ^{his}-*oprM*	This study
NKS1423	Δ*acrB* Δ*rfaC* /pMMB67HE-*mexAB* (F178W)^{his}-*oprM*	This study

Supplementary refference

Horiyama T, Yamaguchi A, Nishino K. TolC dependency of multidrug efflux systems in Salmonella enterica serovar Typhimurium. *J Antimicrob Chemother* 2010; 65: 1372-6.

Supplementary Table S2. Data collection and refinement statistics.

	MexB + LMNG	MexB F178W + LMNG	MexB + C7NG
**Data Collection**			
Space group	*P*1	*P*1	*P*1
Wavelength (Å)	0.9000	0.9000	0.9000
Cell dimensions			
a, b, c (Å)	122.95, 134.35, 149.69	123.36, 134.21, 150.27	125.25, 135.68,152.30
α, β, γ (°)	92.47, 109.80, 89.02	87.24, 70.205, 88.59	86.66, 69.54, 87.84
Resolution (Å)	100-2.90(2.95-2.90)	100-3.15(3.20-3.15)	100-3.65 (3.71-3.65)
R_{merge}	5.7 (75.3)	6.3 (79.4)	12.2 (66.8)
$CC^{1/2}$	N.A. (0.786)	N. A. (0.725)	N.A. (0.683)
$I/\sigma I$	24.3 (2.0)	21.9 (2.0)	14.2 (2.8)
Completeness (%)	98.9 (98.7)	98.8 (98.8)	99.3 (99.2)
Redundancy	3.9 (3.9)	3.8 (3.7)	4.0 (4.0)
**Refinement**			
Resolution (Å)	50-2.90	50-3.15	50-3.65
No. reflections	778,039	575,396	412,035
R_{work}/R_{free}	23.4 /27.8	21.9/29.0	24.7/28.5
R.m.s. deviations			
Bond lengths (Å)	0.013	0.011	0.010
Bond angles (°)	1.662	1.601	1.479

Highest resolution shell is shown in parenthesis.

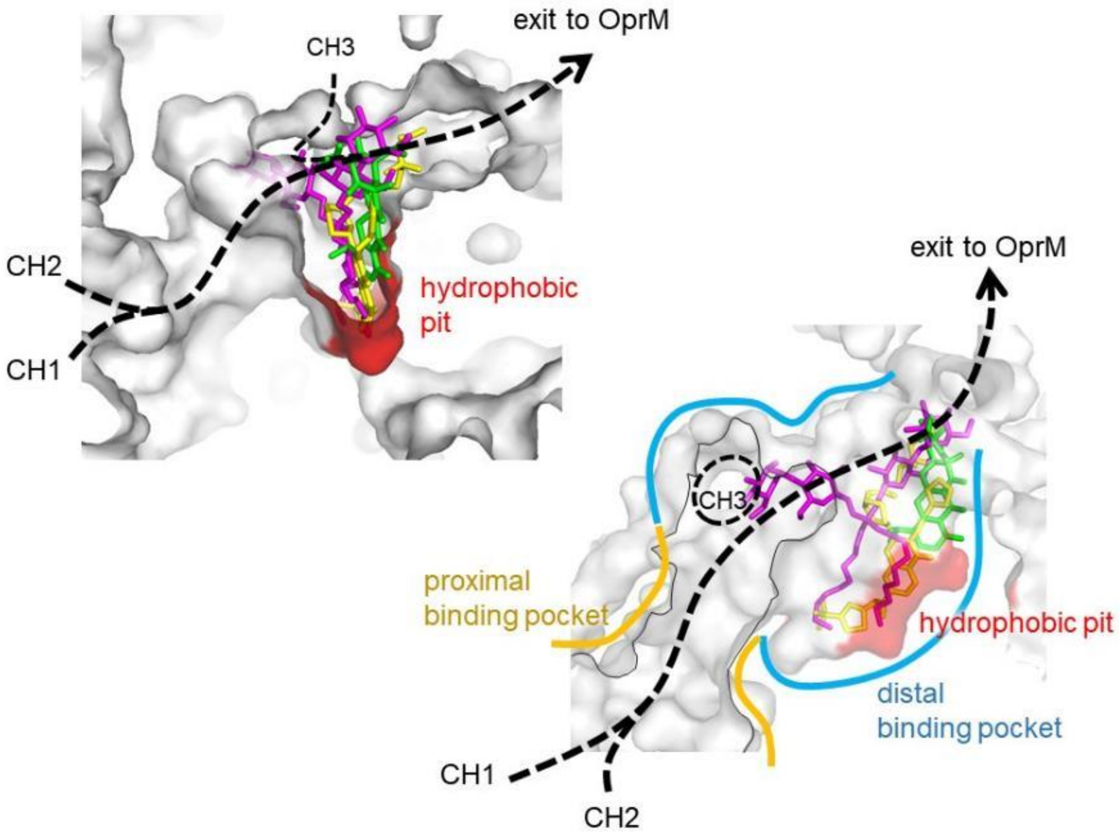

Supplementary Figure S1. Cut view of the substrate translocation channel and the hydrophobic inhibitor-binding pit with bound substrates in the binding monomer. Upper left panel is the side view of the channel from CH1,2 to the exit to the centre funnel-like opening overlapping with bound LMNG (pink), Minocycline (green, PDB ID: 3AOD) and ABI-PP (yellow, PDB ID: 3W9J). Lower right panel is the view rotated from the upper left panel to show the overall picture of the hydrophobic inhibitor-binding pit with bound LMNG. Hydrophobic pit is shown by the red surface. Dotted arrows indicate the putative substrate translocation route.

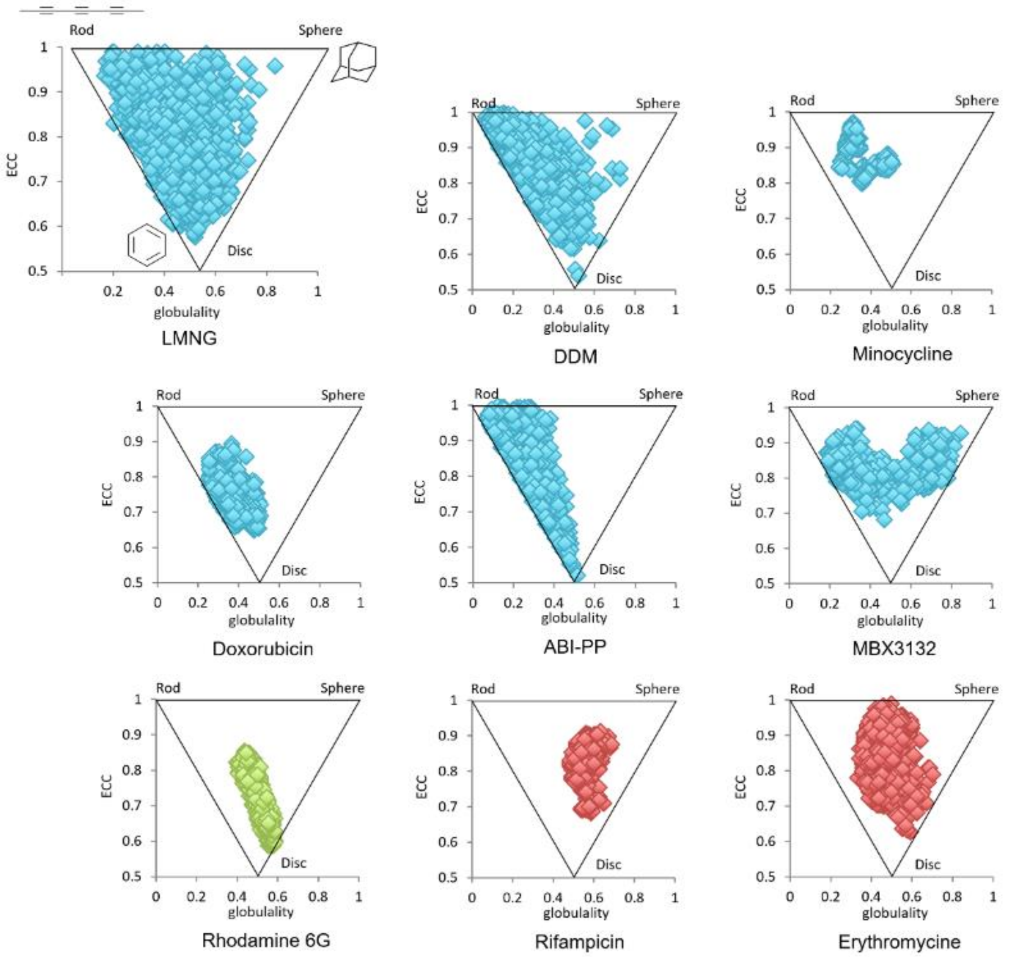

Supplementary Figure S2. Shape diversity analysis (principal moments of inertia (PMI) analysis)[34].

PMI values were calculated, and the normalized PMI value ratio is plotted. Compounds are coloured blue, red and green, showing DBP-binding substrates, rhodamine 6G (a DBP-binding substrate that enters from channel 3[33]), and PBP-binding substrates, respectively. In this figure, compounds observed in physiologically relevant asymmetric crystals were selected.

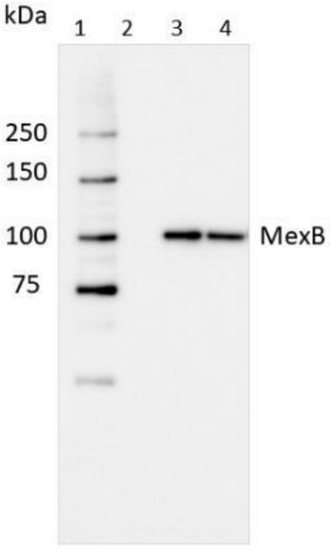

Supplementary Figure S3. Expression level of MexB and its F178W mutant. Membrane fractions (2 μg protein) were separated by SDS-PAGE, and the MexB proteins were visualized by western blot analysis using monoclonal anti-polyhistidine antibodies (MBL code: D291-3, Anti-His-tag mAb). Lane 1: MW marker, 2: vector, 3: MexAB-OprM, 4: MexA-MexB(F178W)-OprM.

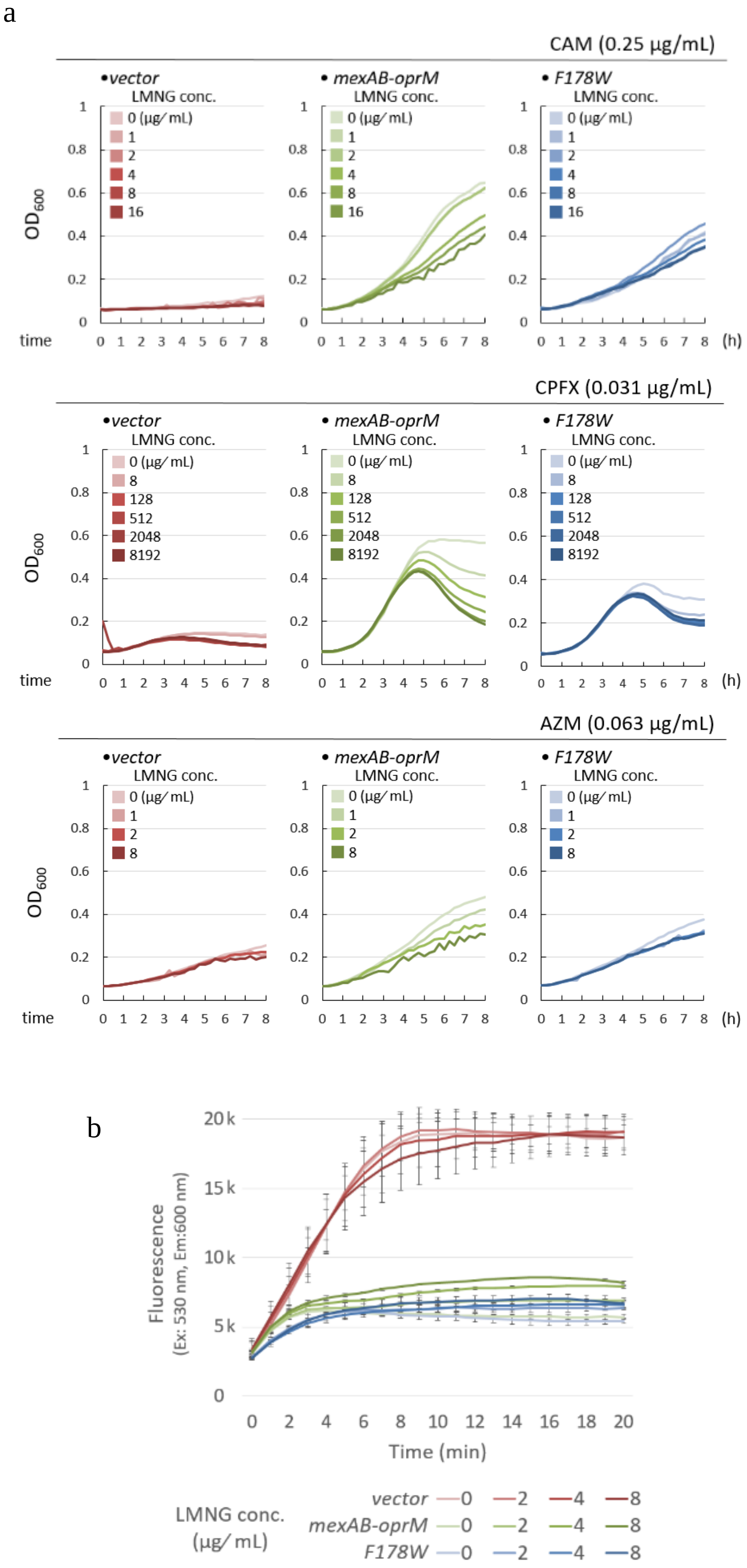
a
CAM (0.25 μg/mL)
•vector
• mexAB-oprM
• F178W
LMNG conc.
0 (μg/ mL)
1
2
4
8
16
OD600
time
(h)
CPFX (0.031 μg/mL)
0 (μg/ mL)
8
128
512
2048
8192
AZM (0.063 μg/mL)
0 (μg/ mL)
1
2
8
b
Fluorescence
(Ex: 530 nm, Em:600 nm)
20 k
15 k
10 k
5 k
0
Time (min)
LMNG conc.
(μg/ mL)
vector —0 —2 —4 —8
mexAB-oprM —0 —2 —4 —8
F178W —0 —2 —4 —8

Supplementary Figure S4. Inhibitory effect of LMNG on MexAB-OprM-mediated drug efflux in the rough mutant of *acrB*-deficient *S. enterica*. (a) The effect of LMNG on the growth of cells in the presence of clarithromycin, ciprofloxacin and azithromycin. Left panel (red): the strain transduced with the vector; central panel (green): the strain expressing MexA-MexB-OprM; right panel (blue): the strain expressing MexA-MexB(F178W)-OprM. (b) The effect of LMNG on the MexAB-OprM-mediated prevention of berberine accumulation in the ΔacrBΔrfaC mutant of S. enterica. Berberine (15 μM) and the indicated amount of LMNG were added.

In the case of ciprofloxacin, the OD value decreases in the latter graph because of its own property. Because it functions as an antibiotic agent by inhibiting DNA gyrase, it takes time to show the sterilization effect arising from the addition.

EM: erythrocin, EtBr: ethidium bromide, CAM; clarithromycin, CPFX: ciprofloxacin, AZM: azithromycin

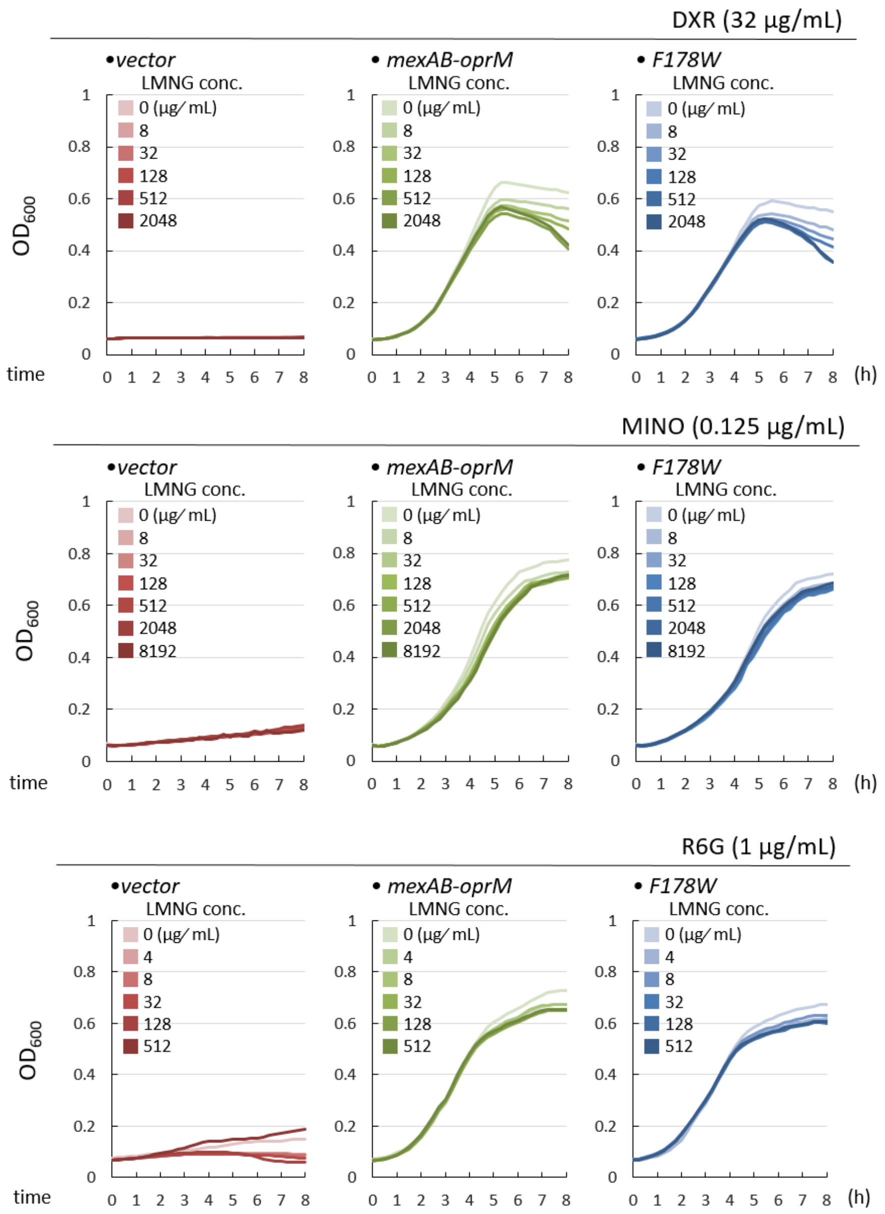

Supplementary Figure S5. Effect of LMNG on MexAB-OprM-mediated drug efflux in the rough mutant of *acrB*-deficient *S. enterica*.

Competitive inhibition against doxorubicin, minocycline, and rhodamine 6G by LMNG in a concentration-dependent manner was not observed. In addition, there was no difference between the wild-type MexB and F178W variants. The colours are the same as in Supplementary Figure S4.

DXR: doxorubicin, MINO: minocycline, R6G: rhodamine 6G

(a) (b)

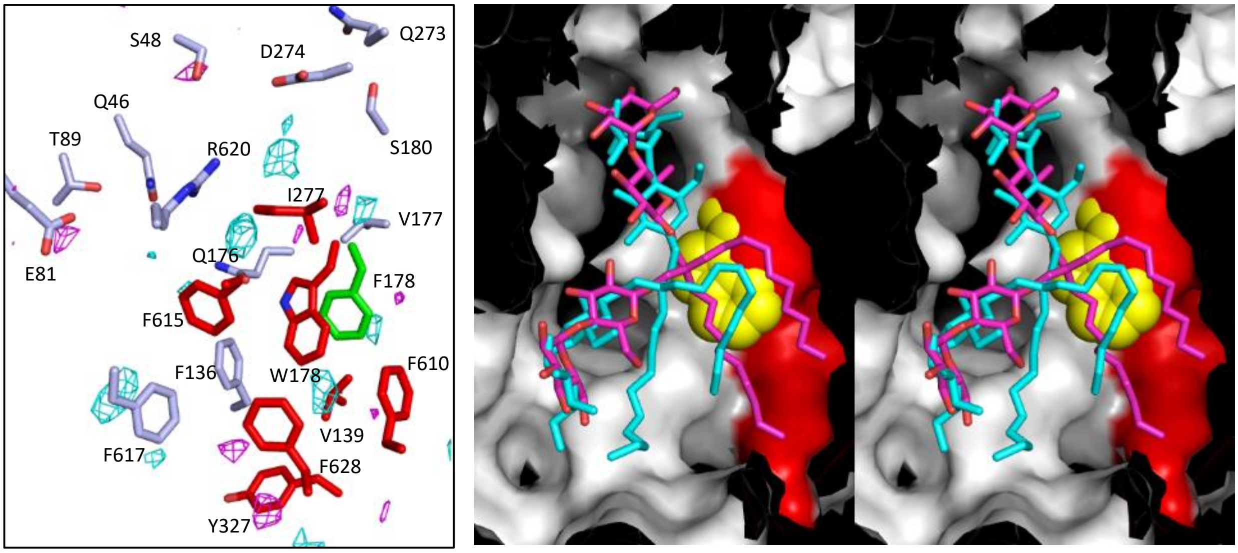

Supplementary Figure S6. Co-crystal structure of MexB(F178W) and LMNG.

(a) Close-up view of the distal binding pocket of MexB(F178W) co-crystalized with LMNG. Carbon atoms of the amino-acid side chains composed of hydrophobic inhibitor-binding pit are shown in red. F178 of the wild-type MexB is shown in green. *F*o-*F*c omit maps contoured at +3.0 σ and -3.0 σ are shown in blue and pink mesh, respectively. The view angle and the range are the same as in Figure 2b. **(b)** Surface cut stereo view of wild-type LMNG-binding MexB overlapped with Trp178 (space-filling model, yellow) and LMNG of the MexB(F178W) mutant. The mutant-bound LMNG is a docking model calculated using Glide (Schrödinger). The direction of the view is the same as Fig.2c. LMNG is shown in pink for wild-type and cyan for mutant, respectively.

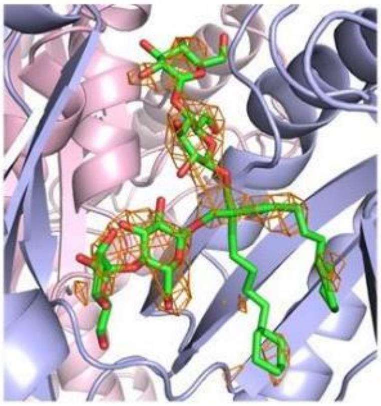

Supplementary Figure S7. Electron density of C7NG calculated as an *F*o-*F*c omit map contoured at 2.5 σ (orange mesh) overlapped with the stick model (green) of C7NG.

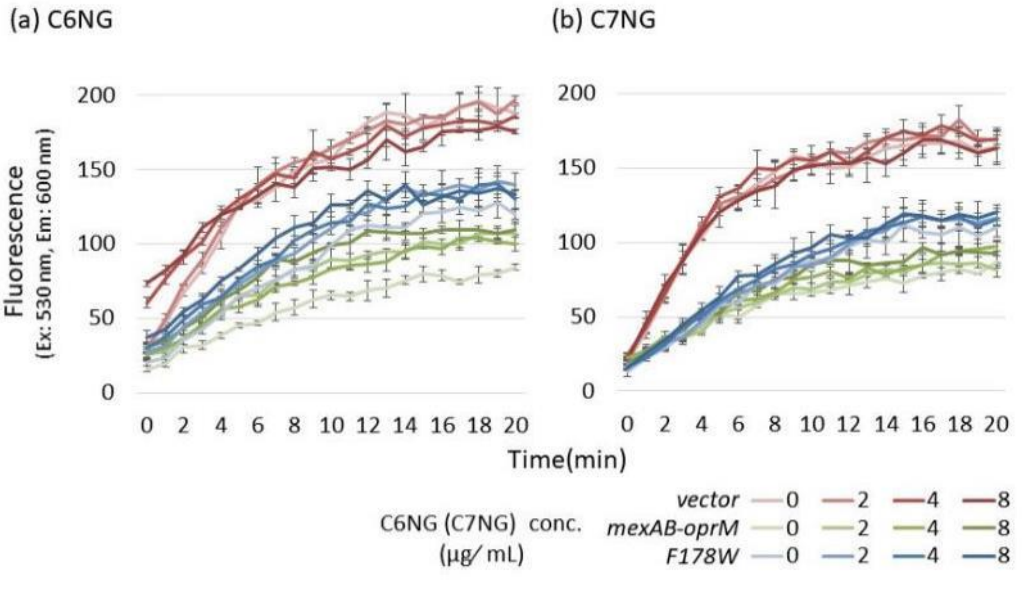

Supplementary Figure S8. The effect of C6NG **(a)** and C7NG **(b)** on the prevention of ethidium bromide accumulation by MexAB-OprM and its F178W mutant. Colours are the same as in Figure 4b.
